# Body mass index and vitamin D level in carpal tunnel syndrome patients

**DOI:** 10.1186/s41983-018-0009-z

**Published:** 2018-05-03

**Authors:** Rania S. Nageeb, Nahed Shehta, Ghada S. Nageeb, Alaa A. Omran

**Affiliations:** Faculty of Medicine, Zagazig University, Sharkia, Egypt

**Keywords:** Vitamin D (25-dihydroxy cholecalciferol), Electrodiagnosis, Body mass index (BMI), Carpal tunnel syndrome

## Abstract

**Background:**

Carpal tunnel syndrome (CTS) is the most common entrapment neuropathy of the upper extremity. The aim of this study is to evaluate the body mass index (BMI) and vitamin D levels in CTS patients.

**Methods:**

The current study was conducted at Zagazig University Hospitals. It included 50 CTS patients and 50 controls. Clinical assessment was carried out to exclude symptoms and signs of neuropathy. Laboratory investigations including vitamin D levels, glycosylated hemoglobin, liver, and kidney function were carried out for every participant. All patients underwent electrodiagnostic study and completed Boston questionnaire to assess their pain sum score, symptom severity (SSS), and functional status (FSS).

**Results:**

Patients had significantly higher BMI and lower vitamin D levels compared to controls (*p* = 0.003 and *p* = 0.001, respectively). Those with severe CTS had a significantly higher BMI and lower vitamin D levels than the others (*p* = 0.03 and *p* = 0.01 respectively). No significant difference was found between CTS subgroups regarding the SSS, while a higher significant FSS and pain sum score were reported in the severe CTS patients compared to the other two groups (*p* = 0.01 and *p* = 0.04 respectively). A significant negative correlation was detected between vitamin D levels and both of BMI, and Boston pain sum scores (*p* = 0.01 and *p* = 0.03 respectively). Also, an inverse correlation was detected between vitamin D levels and both of SSS and FSS (*p* = 0.14, *p* = 0.06). Furthermore, a significant positive and negative correlation between vitamin D levels and both of conduction velocity and distal motor latency respectively was observed (*p* = 0.02 and *p* = 0.01 respectively).

**Conclusions:**

Carpal tunnel syndrome was significantly associated with hypovitaminosis D especially in patients with higher BMI. This highlights the importance of vitamin D supplements and weight loss regimes to minimize the severity of their pain.

## Background

Vitamin D is a steroid molecule, synthesized in the skin from 7-dehydrocholesterol by ultraviolet irradiation or obtained through the diet (Holick 2007). Vitamin D plays a broad range of actions being effective in musculoskeletal system health and calcium-phosphorus metabolism (Hamilton 2010). In addition, data from experimental studies provided evidence that vitamin D has a role in neuroprotection and neurotrophism (DeLuca et al. 2013) as it was reported that its administration reduced neurological injury and neurotoxicity in various animal brain systems (Brewer et al. 2001) and improved myelination and recovery after nerve injuries (Chabas et al. 2013).

Low vitamin D is related to neuropathy in patients with diabetes and other neurodegenerative disorders (Shehab et al. 2012; Moon et al. 2015). Also, vitamin D deficiency has been reported to play a potential role in non-specific persistent painful conditions (Kuru et al. 2015).

Carpal tunnel syndrome (CTS) is the most common entrapment neuropathy of the upper extremity that results in significant functional disability (Miyamoto et al. 2016). CTS has a multifactorial etiology including systemic, anatomical, and idiopathic causes. This type of entrapment neuropathy occurs due to increased pressure in the carpal tunnel on the median nerve inducing marked changes in intraneural microcirculation and nerve fiber structure, impairment of axonal transport, and alterations in vascular permeability, with edema formation and deterioration of nerve function (Aboonq 2015).

Some parameters such as age, gender, and body mass index (BMI) could be a risk factor for CTS. The association between increased BMI and CTS has been studied previously (Lam and Thurston 1998; Komurcu et al. 2014), and the high risk of developing CTS in the obese individuals (BMI > 30) was argued to increased fat deposition in the carpal canal and elevated hydrostatic pressure over the median nerve within the carpal tunnel in the obsess subjects (Komurcu et al. 2014).

A study on proteome alterations in serum found that vitamin D-binding proteins were downregulated in patients with CTS (Oh et al. 2013). Moreover, vitamin D had been reported to be a suppressing factor for vascular endothelial growth factor (VEGF) (Gruber et al. 2008; Hirsch et al. 2011), which was increased in the synovial biopsy of patients with CTS (Hirata et al. 2004). The increased level of VEGF was associated with increased vascular proliferation and inflammatory synovial fibrosis that could trigger the occurrence of CTS. Based on these observations, researchers (Donato et al. 2009) had stated that non-surgical management of carpal tunnel syndrome might be conducted via anti-VEGF agents; one of them was the vitamin D.

The aim of the present study was to assess the body mass index (BMI) and serum levels of vitamin D in CTS patients and to examine if a possible association could be found between vitamin D and electrophysiological data, symptom and pain severity, and functional status in CTS.

## Methods

The present study was a case-control type. Patients were selected from attendee of out-patient Neurology and Rheumatology and Rehabilitation clinics of Zagazig University Hospitals, Sharkia Governorate, Egypt in the period from September 2016 to March 2017. Samples were collected by the systematic random method.

### Sample size calculation

A sample size of 33 patients and 33 controls was found to provide 80% power, at 0.05 alpha level of significance. We used a sample of 50 CTS patients and 50 controls to increase the power of our study. Epi Info version 7 was used for the calculation (CDC 2015).

Two main groups were included in this study: Carpal tunnel syndrome (CTS) group consisted of 50 adults presented with clinical and electrophysiological evidence of CTS (35 females and 15 males). Their mean age was 42.3 ± 11.8 (range 29–45 years).

### Control group

Control group consisted of 50 (30 females and 20 males) volunteers, free of clinical and electrophysiological evidence of CTS. We enrolled healthy subjects from the relatives of patients during the same study period consecutively, without complaints of numbness or tingling in the median nerve distribution, they were age and sex matched with the patients, and their age mean (±SD) was 40.4 (± 12.9) years.

### Ethical consideration

An informed consent was taken from all of the participants after explaining the details, benefits, and risks to them. This study was approved by the local ethical of Faculty of Medicine, Zagazig University.

### Inclusion criteria

Patients were eligible for inclusion in this study if they had clinical and electrophysiological evidence of CTS according to Wang (2013): Evidence of CTS characterized by either or both the following:

- Median nerve peak sensory latency > 3.5 ms (stimulated at wrist at distance from active electrode = 13 cm)
- Median nerve distal motor latency > 4.4 ms (stimulated at wrist at distance from active electrode = 7 cm)

### Exclusion criteria

Subjects were excluded if any of the following was detected:

- History, clinical signs, or electrodiagnostic findings suggesting coexisting neurological conditions (e.g., polyneuropathy, hereditary neuropathy)
- Sub-clinical sensory polyneuropathy.
- History or clinical signs suggesting coexisting rheumatological disease.
- Symptoms, signs, or radiological findings of cervical radiculopathy in association with CTS.
- Symptoms or signs suggesting systemic clinical illness influencing mineral metabolism, like diabetes mellitus, hyperthyrodism, hyperparathyroidism, inflammatory thyroiditis, malignancy, inflammatory arthritis, renal failure, and hepatic failure.
- Previous surgery or trauma involving the upper limb and/or neck.
- Subjects currently receiving calcium/vitamin D supplementation.
- Patients of renal dialysis.

All patients included in the present study are subjected to:

1. Full history taking.
2. Thorough general and neurological examination, for diagnosis of CTS and exclusion of polyneuropathy that are associated with peripheral nerve entrapment (Cranford et al. 2007; Uchiyama et al. 2010).
3. Body mass index (BMI) was calculated by dividing body weight in kilograms by height in meters squared. BMI was divided into the following categories: normal weight < 25 kg/m^2^, overweight 25– < 30 kg/m^2^, class 1 obesity 30 to < 35 kg/m^2^, and class 2 obesity ≥ 35 kg/m^2^ (Garrow and Webster 1985).
4. Routine laboratory tests for the exclusion of other systemic affection like diabetes mellitus and renal or hepatic failure.
5. Estimation of serum vitamin D levels
  Fasting blood samples were collected from patients and controls for serum vitamin D assays. The blood sampling of patients for serum vitamin D assays was performed in the same day of electrophysiological examination and of controls in the same months in study period. Serum was collected using standard sampling tubes or tubes containing separating gel. 25(OH) Vitamin D is stable for 8 h at 18–25 °C, 4 days at 2–8 °C, and 24 weeks at − 20 °C). Vitamin D was determined by the radioimmunoassay method using 25-OH Vitamin D EIA Kit (Immundiagnostik, Bensheim and Biomedica, Wien). Patients with vitamin D ˂ 20 ng/ml were considered to have vitamin D deficiency and those between 20.0 and 30.0 ng/ml as “insufficient,” while optimal levels were defined as vitamin D concentrations greater than 30.0 ng/ml (Ozkan et al. 2012).

### Electrodiagnostic studies (Wang 2013; Roll et al. 2011)

All tests were done in the same room, using a NIHON KOHDEN CORPORATION (1–31-4 Nishiochiai, Shinjuku-Ku, Tokyo, Japan). The computer used accepts voltage ranges from 220 to 240 V and 50 to 60 Hz. The electrodiagnostic studies took place at the time of clinical diagnosis. Upper extremity temperature was maintained at or above 30 °C at time of examination asking our subjects to maintain their hands in the pockets of their clothes or putting hands in the container of warm water. The electrophysiological studies included the following:

#### Motor nerve conduction studies

Motor nerve conduction study of the median nerve was done recording as usual from abductor pollicis brevis (APB) muscle while stimulating the median nerve at wrist and elbow sites. Distal latency, compound muscle action potential (CMAP), and forearm motor conduction velocities were recorded (normative values: distal latency ≤ 4.4 ms, CMAP amplitude ≥ 4.0 mV, conduction velocities ≥ 49 m/s).

#### Sensory nerve conduction studies

Antidromic median sensory recording over digit 2 while stimulating the median nerve 13 cm proximal to the active electrode. Sensory peak latency and sensory nerve action potential (SNAP) amplitude were measured (normative values: peak latency ≤ 3.5 ms, SNAP amplitude ≥ 20 mV).

#### Electromyographic study

Electromyographic study (EMG) was done using disposable concentric needles for abductor pollicis brevis as well as proximal median muscles (pronator teres, flexor pollicis longus) to localize entrapment at wrist. Sampling of extensor indicis and abductor digiti minimi (muscles supplied by C8 root, and not belong to median nerve as well as cervical paraspinal muscles to exclude radiculopathy and polyneuropathy (Cranford et al. 2007).

### The severity of CTS according to the electrophysiological data was scored according to Watson (2012)

Mild CTS is prolonged sensory peak latencies (> 3.5 ms) ± amplitude reduction (< 20 μV) with normal motor studies, and no evidence of axon loss. Moderate CTS is abnormal median sensory latencies as noted for mild CTS plus prolongation of median motor distal latency (> 4.4 ms), with no evidence of axon loss. Severe CTS is any of the aforementioned NCS abnormalities with evidence of axon loss as defined by either (1) an absent or low-amplitude SNAP or mixed nerve action potential; (2) a low-amplitude (< 4.0 mV) or absent thenar muscles CMAP; or (3) a needle EMG with fibrillation potentials or motor unit potential changes (large amplitude, long-duration motor unit potentials, or excessive polyphasics) in the muscles supplied by the median nerve.

### We assessed symptom severity of carpal tunnel syndrome and patient functional status using the Boston CTS questionnaire (Levine et al. 1993)

The questionnaire, designed to be completed independently by the patient, is in two sections: the symptom severity scale consists of 11 questions with multiple-choice responses, scored from 1 point (mildest) to 5 points (most severe); the functional status scale consists of 8 questions with multiple-choice responses, scored from 1 point (no difficulty with the activity) to 5 points (cannot perform the activity at all). Levine et al. (1993) stated that means and standard deviations should be used to calculate the symptom and function scores. Thus, a higher score indicates worse symptoms or dysfunction. Furthermore, the sum score of the first five questions of the Boston questionnaire symptom severity scale was calculated to evaluate to severity of pain.

### Statistical analysis

Descriptive statistical methods were used to calculate means and SDs. For comparisons with the continuous variables, Student’s *t* test as well as Anova was used. Comparison of categorical data was performed using the *χ*^2^ test and the Fisher exact test. Data were analyzed using statistical package of social science, version 14.0.0 software package (Levesque 2007).

## Results

Fifty patients (15 males and 35 females) clinically and electrophysiologically proved as having CTS beside the fifty (20 males and 30 females) controls were included in the present study. Their mean ages (±SD) were 42.3 (± 11.8) and 40.4 (± 12.9) years respectively.

Body mass index (BMI) was significantly higher in CTS patients compared to controls (*p* = 0.003). CTS patients had significantly lower vitamin D levels compared to controls (*p* = 0.001) (Table 1).

**Table 1:** Comparison between CTS patients and controls regarding the demographic and laboratory data

Variable	CTS group *N* = 50	Controls *N* = 50	*p* value
Age (years)	42.3 ± 11.8	40.4 ± 12.9	0.14
Sex, *N* (%)			
Female	35 (62.2%)	30 (60%)	0.68
Male	15 (37.8%)	20 (40%)	
Body mass index (kg/m^2^)	31.9 ± 5.1	23.3 ± 2.3	0.003*
Serum vitamin D (ng/ml)	19.1 ± 6.2	30.4 ± 10.9	0.001*

Continuous data are represented in mean ± SD; categorical data are represented in number and percentage

*CTS* carpal tunnel syndrome

*Significant

We observed that 17 (34%) patients had mild entrapment, 19 (38%) had moderate entrapment, and 14 (28%) had severe entrapment. There was significant difference in the BMI between CTS subgroups, *p* = 0.03 (those with severe CTS had a higher BMI than the others). Vitamin D levels were significantly lower in patients with severe CTS compared to mild, and moderate entrapment (*p* = 0.01) (Table 2).

**Table 2:** Relation of carpal tunnel syndrome severity (according to electrophysiological staging) and body mass index as well as vitamin D levels

Electrophysiological severity of CTS patients	BMI	Vitamin D levels
Mild 17 (34%)	27.25 ± 4.3	24.3 ± 7.2
Moderate 19 (38%)	28.43 ± 5.1	20.5 ± 6.8
Severe 14 (28%)	32.74 ± 5.4*	12.6 ± 4.5*
*p* value	0.03	0.01

Quantitative data are represented in mean ± SD

*CTS* carpal tunnel syndrome, *BMI* body mass index

*Significant

No significant difference was found between the CTS subgroups regarding the Boston Symptom Severity score, while a higher functional status score and pain sum score were significantly reported in the severe CTS patients compared to the other two groups (*p* = 0.01 and *p* = 0.04 respectively) (Table 3).

**Table 3:** Relation of Boston questionnaire scores and electrophysiological severity of carpal tunnel syndrome

Electrophysiological severity of CTS patients	Boston Questionnaire Scores		
	Symptom severity score	Functional status score	Pain sum score
Mild	2.63 ± 0.93	2.29 ± 0.97	12.47 ± 6.85
Moderate	2.84 ± 0.73	2.30 ± 0.95	13.75 ± 4.57
Severe	2.99 ± 0.80	4.31 ± 0.91*	17.21 ± 3.50*
*p* value	0.51	0.01	0.04

Data are represented in mean ± SD

*CTS* carpal tunnel syndrome

*Significant

A significant negative correlation was detected between vitamin D level and both of BMI, and Boston pain sum scores (*p* = 0.01 and *p* = 0.03 respectively). Also, an inverse correlation was seen between vitamin D levels and both of Boston symptom severity and functional status scores (*p* = 0.14, 0.06) but it did not reach a significant level. Furthermore, a significant positive correlation between motor conduction velocity and level of vitamin D was observed (*p* = 0.02). A significant negative correlation was also found between distal motor latencies and vitamin D level (*p* = 0.01). Also, an inverse correlation was seen between vitamin D levels and peak sensory latencies but it did not reach a significant level (*p* = 0.24) (Table 4).

**Table 4:** Correlation coefficient of variables with serum levels of vitamin D

Variable		Vitamin D level (ng/ml)	
		*R*	*p* value
Body mass index		− 54	0.01*
Boston Questionnaire Scores	Pain sum score	− 43	0.03*
	Symptom severity score (SSS)	− 17	0.14
	Functional status score (FSS)	− 21	0.06
Median nerve electrophysiological parameters	Compound muscle action potentials amplitude	+ 17	0.73
	Motor conduction velocity	+ 35	0.02*
	Distal motor latency	− 53	0.01*
	Sensory nerve action potentials amplitude	+ 11	0.55
	Median nerve peak latency	− 13	0.24

*Significant

## Discussion

There is growing evidence suggesting that the role of vitamin D is not confined to calcium and phosphate homeostasis only but also there has been interest among researchers to identify other target organs affected by vitamin D especially peripheral nervous system (Shehab et al. 2012). As CTS is considered as the most common entrapment neuropathy, we conducted this study searching for a possible association between CTS on the one hand and BMI as well as vitamin D levels on the other hand.

The present study showed that CTS patients had a significantly low vitamin D levels than controls. This observation was in agreement with recent studies (Tanik et al. 2016). Similarly, in their study, Gursoy et al. (2016) observed significantly low vitamin D level in patients with CTS compared to controls.

In line with the present study that support the role of vitamin D in peripheral nerve function, data from previous studies which were conducted on a group of patients with diabetic neuropathy showed a relevant association between vitamin D deficiency and the development of diabetic neuropathy (Skalli et al. 2012; Soderstrom et al. 2012; Putz et al. 2013). Similarly, Celikbilek et al. (2015) observed in their study of diabetics that patients with neuropathy had lower levels of vitamin D compared to those without. Also, hypovitaminosis D has also been reported in patients with nonspecific painful conditions (Plotnikoff and Quigley 2003) and in patients with chronic low back pain (Al Faraj and Al 2003).

Vitamin D is considered as a neuroactive steroid; it was recorded previously that vitamin D induces nerve growth factor and hence could help in the prevention of neurotrophic deficits (Riaz et al. 1999). Furthermore, Chabas et al. (2013) demonstrated that vitamin D influences the myelination through the activation of several myelin-associated genes.

BMI is a good indicator of body fat (Kouyoumdjian et al. 2002). Various studies had suggested that obesity increases the risk of carpal tunnel syndrome (Lam and Thurston 1998; Komurcu et al. 2014). Moreover, Komurcu et al. (2014) observed in their study that as the BMI increased, the severity of CTS does in a significant manner. These data support our finding that CTS group had a significantly higher body mass index than controls. This was explained as in obese individuals, there is increased fat deposit and hydrostatic pressure in the carpal tunnel (Komurcu et al. 2014).

In regard to the CTS electrophysiological severity, this study demonstrated a significant difference between mild, moderate, and severe CTS patients in respect to BMI, being the highly recorded BMI was in patients with severe CTS. In addition, an inverse correlation was seen between BMI and vitamin D levels in this study which was concordant with the finding of Werner et al. (1994) and Wortsman et al. (2000) who found that obesity was significantly associated with lower vitamin D levels. It has also been suggested that the metabolic clearance of vitamin D might be increased in obesity, possibly with enhanced uptake by adipose tissue and, consequently, alteration of its release to the circulation (Werner et al. 1994).

Data regarding association between the electrophysiologic severity of CTS and vitamin D level is deficient. In this study, we assessed if a relation could be found between vitamin D levels and the electrophysiologic severity of CTS. As we classified the CTS patients according to the electrophysiologic severity into mild, moderate, severe groups, the lowest vitamin D levels were observed in patients with severe CTS. Tanik et al. (2016) demonstrated that the severity of vitamin D deficiency was associated with CTS severity which was matching with our results. Different from ours, Gursoy et al. (2016), who studied patients with CTS and classified them into electrophysiologically confirmed and electrophysiologically negative groups, found no significant relation between severity of CTS and vitamin D levels in their cohort.

On studying the relation between selected nerve conduction study parameters, we observed a significant positive correlation between motor conduction velocity and vitamin D levels, while a negative correlation was seen between distal motor latency and vitamin D. Our results are supported by McDermott et al. (2012) who found a significant association between low vitamin D level and the slowing of conduction velocities. Furthermore, Kuru et al. (2015) recorded an inverse correlation between vitamin D level and motor distal latencies. Moreover, Shehab et al. (2015) observed a direct relationship between vitamin D level and both of nerve conduction velocity and CMAP amplitudes of all examined nerves in their prospective study of patients with diabetic neuropathy who underwent short term vitamin D supplementation. In addition, data from animal studies showed that hypovitaminosis D was associated with induced nerve conduction abnormalities. Consistent with these results, basic research studies and animal data demonstrated that vitamin D receptors exist on peripheral nerves and Schwann cells, and consequently, vitamin D could promote production of nerve growth factor and axon regeneration in peripheral nerves (Neveu et al. 1994; Cornet et al. 1998; Chabas et al. 2008).

On assessment of symptom and pain severity as well as functional status of our CTS group, we utilized the Boston questionnaire (Levine et al. 1993) with its two subsets. No significant difference was found between the CTS subgroups in regard to the symptom severity, while a higher pain severity and functional status impairment were found in the severe CTS group than in the other two groups. Also, regarding the relation between vitamin D levels and the Boston questionnaire results, an observed inverse correlation was seen between vitamin D levels and pain sum scores, while we could not observe a similar significant relation between vitamin D and either symptom severity or functional status scores.

The present findings met with the findings of another study conducted on diabetic patients demonstrated that neuropathic pain scores were inversely correlated with the levels of vitamin D; also they concluded that vitamin D deficiency was an independent risk factor for neuropathy (Celikbilek et al. 2015). In addition, Lee and Chen (2008) found that conservative vitamin D supplementation for 3 months (2000 IU/day) resulted in a decrease of 50% in the pain score in patients with type 2 diabetes and associated chronic pain neuropathies. They suggested that a vitamin D deficiency may contribute to neuropathic pain and that vitamin D supplements may be an effective analgesic treatment modality. Furthermore, vitamin D deficiencies have been associated with a lower pain threshold, which shows a non-specific increase after correcting for the vitamin D deficiency (Plotnikoff and Quigley 2003).

The significant relation between increased pain severity and low vitamin D levels could be explained by the reduced anti-inflammatory activity of vitamin D through the regulation of interleukin, tumor necrosis factor, and macrophage activity (DeLuca et al. 2013). In addition, hypovitaminosis D also may be resulted in hyperinnervation and hypersensitivity in nerve fibers which are carrying pain sensation and cause increased pain perception in already existing pathology (Tague et al. 2011).

## Conclusions

Carpal tunnel syndrome (CTS) patients had a lower level of vitamin D and higher BMI comparable to controls. In addition, pain severity was highly related to low vitamin D level in CTS sufferers. Based upon, CTS patients especially obese subjects could be recommended to receive vitamin D supplements and undergo weight reduction regimes as these modalities could minimize the severity of pain. So, further studies are recommended to verify whether hypovitaminosis D correction could be of benefit in treating and reducing pain severity in CTS patients.

## Abbreviations

APB: Abductor pollicis brevis
BMI: Body mass index
FSS: Functional status score
SSS: Symptom severity score
CMAP: Compound muscle action potential
CTS: Carpal tunnel syndrome
EMG: Electromyographic study
SNAP: Sensory nerve action potential
VEGF: Vascular endothelial growth factor
